# The Impacts of Dengue Virus Infection on Mitochondrial Functions and Dynamics

**DOI:** 10.3390/ijms26188968

**Published:** 2025-09-15

**Authors:** Showkot Ahmed, Réka Dorottya Varga, Jinsung Yang

**Affiliations:** Department of Biochemistry and Convergence Medical Science, Institute of Medical Science, College of Medicine, Gyeongsang National University, Jinju 52727, Republic of Korea

**Keywords:** dengue virus, mitochondria, mitochondrial bioenergetics, mitochondrial dynamics, cellular homeostasis, mitophagy, biogenesis

## Abstract

Dengue virus (DENV) is a mosquito-borne flavivirus responsible for a significant global disease burden, especially in tropical and subtropical regions. DENV critically manipulates host cell mitochondria to ensure its replication and survival. The clinical manifestations are well-studied and how dengue infection significantly alters the mitochondrial dynamics, and the subsequent functional cellular homeostasis has been unveiled. This review discusses the strategies by which DENV alters mitochondrial functions and dynamics. It particularly focuses on the virus-induced suppression of mitochondrial quality control mechanisms like mitophagy. Moreover, the dichotomous role of mitophagy in supporting DENV replication is highlighted. By incorporating recent studies about DENV-host interactions at the mitochondrial interface, mitochondria, as regulators and targets in dengue pathogenesis, are suggested as possible molecular targets for therapeutic intervention.

## 1. Introduction

Dengue is an acute febrile mosquito-borne viral disease seen in tropical and subtropical regions, causing a significant global health threat. Annually, an estimated 390 million dengue infections occur worldwide, and expanding epidemics now place approximately 3.9 billion people at risk [1,2]. This modern urban transmission cycle was preceded by a deep evolutionary history. The four distinct DENV serotypes originated from an ancestral virus maintained in a sylvatic cycle involving non-human primates in Southeast Asia. The divergence into modern serotypes occurred after these primate populations became geographically isolated on islands due to rising sea levels during the Holocene [3,4].

The early history of dengue involves outbreaks of a dengue-like syndrome that appeared almost simultaneously across three continents in the late 18th century [5]. Given the similarity of symptoms, it is now believed that some of these historical epidemics may have been caused by the Chikungunya virus. The first well-documented clinical accounts of this illness date to 1779, with reports in Asia and North America. It was in Philadelphia that physician Benjamin Rush described the 1780 epidemic, coining the term “break-bone fever” due to the severe musculoskeletal pain. While the Swahili term “Ki pepo denga” (the dengue spirit) exists, the modern name “Dengue” is more commonly thought to have been adopted from the Spanish word dengue, meaning ‘fastidious’ or ‘careful,’ likely referencing the cautious gait of patients suffering from joint pain. The global spread of both the viruses and their mosquito vectors was facilitated by increased maritime commerce and human movement, including the transatlantic slave trade. However, virological proof of dengue fever was not established until the 20th century [5,6,7,8]. A critical period for this research occurred during the Second World War, when widespread outbreaks in places like India, Hawaii, and the Pacific Islands prompted intensive study. It was during this time that researchers, most notably Albert Sabin and his colleagues, made foundational discoveries by isolating and characterizing the dengue viruses [9,10].

DENV is the single-stranded enveloped RNA virus belonging to the genus flavivirus of the family Flaviviridae, which comprises three structural proteins and seven non-structural (NS) proteins. It comprises four antigenically distinct serotypes (DENV-1/4), with a fifth sylvatic serotype (DEMV-5) recently identified in Malaysia, highlighting the ongoing genetic recombination and natural selection. By dint of phylogenetic analysis, DENV-5 shares genetic similarities with the other four serotypes, suggesting a shared evolutionary origin [2,8,11,12,13]. Dengue virus causes a wide range of illnesses, from asymptomatic or mild and self-limiting fever to severe illness along with dengue fever (DF), dengue hemorrhagic fever (DHF), and dengue shock syndrome (DSS) [2,14]. Most dengue virus infections in susceptible adults result in dengue fever, which is a brief, severe, and incapacitating feverish disease [15]. Dengue hemorrhagic fever (DHF) is a severe form of dengue along with blood vessel rupture, first reported in the Philippines in 1953 [6,16]. On the other hand, dengue shock syndrome (DSS) is the fatal manifestation of dengue with substantial plasma losses and hypovolemic shock, considered as a clinical spectrum of DHF by early reports. To date, DSS has been identified as a significant cause of pediatric mortality in endemic areas, especially in Vietnam and Thailand [11,17,18].

Pathologically, DENV infects dendritic cells, macrophages, endothelial cells, and then spreads through the lymphatic vessels to lymph nodes and peripheral blood [19]. After viremia occurs, it triggers a significant innate immune response, which can lead to a cytokine storm. The cytokine storm is responsible for severe clinical outcomes like vascular leakage, thrombocytopenia, and coagulopathy [20,21,22]. To fuel its replication and navigate this adverse host environment, DENV must profoundly alter cellular functions, with recent evidence pointing directly to severe mitochondrial damage (Table 1) [23,24].

Mitochondria, the power generators of cells, are dynamic cytoplasmic organelles. Far more than simple energy producers, they act as central hubs for numerous physiological processes [35,36]. The primary and vital function of mitochondria is to generate energy in the form of ATP through the process of oxidative phosphorylation (OXPHOS), a process intrinsically linked to the controlled production of ROS [37]. As a metabolic center, mitochondria integrate and coordinate a variety of metabolic pathways like the Krebs cycle, fatty acid β-oxidation, OXPHOS, and amino acid metabolism, which are necessary for energy production and biosynthesis [37,38].

Furthermore, mitochondria are a key signaling platform [39]. They regulate intracellular calcium (Ca^2+^) homeostasis and act as gatekeepers of apoptosis, determining the destiny of cells [40]. Crucially, they play a role in innate immunity through the MAVS proteins [41]. Mitochondria, as power generators and key signaling platforms, are a prime target for viral manipulation. Recent research indicates that viral infections influence mitochondrial functions to meet the high energy demands of viral replication [42,43].

DENV infection manipulates ATP production, leading to increased oxidative stress and disrupted mitochondrial fission and fusion [44,45]. Impaired mitochondrial dynamics have an aftermath of fragmented mitochondria, which are responsible for increased ROS production and downregulation of ATP production, further contributing to cellular stress [46]. Such damaged mitochondria would be selectively removed in healthy cells through mitophagy [47]. However, DENV infection has been shown to inhibit this critical quality-control pathway, allowing damaged mitochondria to accumulate. This inhibition of mitophagy exacerbates cellular dysfunction, enhances cell death, and likely contributes to the tissue damage observed in severe dengue cases [24,48].

The strategic manipulation of mitochondria is emerging as a key feature of dengue pathogenesis [49]. Understanding this virus–host interplay is critical for developing new therapeutic approaches [50]. From the above stated aspects, this review aims to (a) investigate the molecular mechanisms by which dengue virus interacts with components of the mitochondria to alter cellular metabolism, and (b) evaluate the impact of DENV on mitochondrial dynamics (fission, fusion, and mitophagy) and mitochondrial bioenergetics (ATP production, respiration) [23]. By integrating the current knowledge, this review will illuminate how DENV systematically hijacks mitochondrial function to drive its replication and disease progression [51].

## 2. DENV-Induced Perturbation of Mitochondrial Dynamics

**Figure 1:**
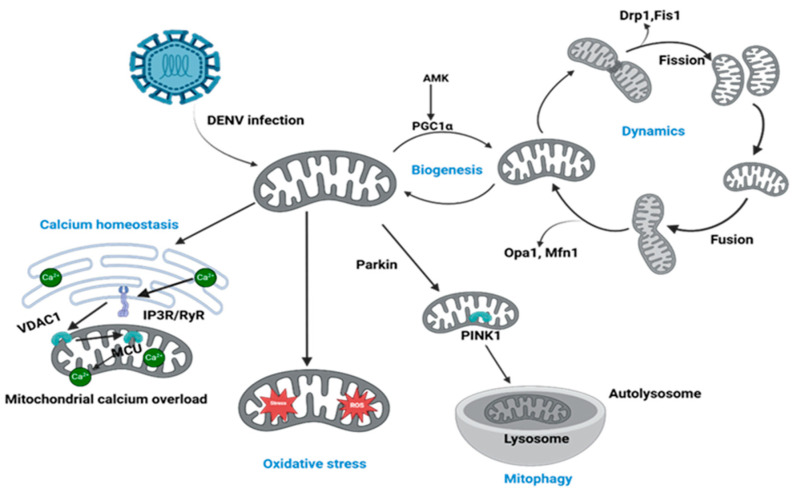
DENV alters mitochondrial dynamics, biogenesis, calcium homeostasis, and mitophagy. DENV induces disruptions in calcium homeostasis by promoting calcium influx via VDAC1 and IP3R/RyR channels, enhancing mitochondrial calcium overload. Mitochondrial biogenesis is dominated through AMPK/PGC1α downregulation; at the same time, mitochondrial dynamics are altered via disproportional fission (Drp1, Fis1) and fusion (OPA1, Mfn1) processes. Further, damaged mitochondria accelerate PINK1/Parkin-mediated mitophagy, contributing to the clearance of dysfunctional mitochondria via lysosomal degradation.

Mitochondrial dynamics illustrate the ongoing processes of fusion, fission, transport, and mitophagy that care for mitochondrial quantity, quality, shape, and functions in cells (Figure 1) [43]. DENV infection severely alters mitochondrial fusion and fission, manipulating several mitochondrial proteins involved in the process [52,53]. Mitochondrial fusion proteins, Mitofusin 1 (MFN1) and Mitofusin 2 (MFN2), are primarily responsible for mitochondrial fusion along with tethering power. The DENV NS2B3 protease complex directly targets and cleaves the mitochondrial fusion proteins MFN1 and MFN2, thereby inhibiting mitochondrial fusion and promoting fragmentation of the mitochondrial network [52]. Dengue virus also affects the mitochondrial fission process by decreasing phosphorylation of dynamin related protein 1 (DRP1) at serine 616 (Ser 616), which is the key protein for mitochondrial fission. This reduces the phosphorylation results in excessive mitochondrial elongation, which is caused by inhibiting mitochondrial fission [53].

However, DENVs have dichotomous roles in the fission process. Dengue viral infection also inhibits mitochondrial fission proteins such as DRP1, which induces mitochondrial elongation along with defective mitochondrial dynamics [53]. DENV infection paradoxically inhibits mitochondrial biogenesis, the pathway responsible for creating new mitochondria [49]. This suppression prevents the replenishment of healthy organelles, thereby exacerbating the accumulation of mitochondrial damage within the infected cell. Dengue virus disrupts mitochondrial biogenesis process by downregulating its master regulators, Peroxisome proliferator-activated receptor gamma coactivator 1-alpha (PGC-1α) and Peroxisome proliferator-activated receptor gamma (PPAR-γ). Since the coordinated activity of the master regulators is essential for driving mitochondrial biogenesis, the DENV-induced downregulation of these proteins critically disrupts this process. As a result, the cell’s capacity to replenish its mitochondrial population is severely compromised, leading to reduced mitochondrial turnover [24]. As PPAR-γ and PGC-1α crosstalk plays a significant role in mitochondrial biogenesis along with PGC-1α transcription and enhanced thermogenesis via uncoupling protein 1(UCP1) activation, dengue-induced downregulation of these two controllers drastically hampers biogenesis [24,54].

## 3. Compromised Mitochondrial Quality Control: The Blockade of Mitophagy and Biogenesis

Mitophagy plays a vital role in cellular maintenance by functioning as a quality control system that removes damaged mitochondria through a targeted autophagy pathway. By selectively eliminating damaged mitochondria, mitophagy serves as a critical quality control mechanism that prevents the downstream consequences of mitochondrial dysfunction. This process preempts the accumulation of excess reactive oxygen species (ROS) and prevents the rupture of the mitochondrial outer membrane, thereby blocking the release of pro-apoptotic factors like cytochrome c and the subsequent activation of caspase-for its replication. This induction of autophagy promotes the formation of mediated apoptosis [47,55,56]. Thus, mitophagy triggers the cellular adaptation to stress and helps maintain energy efficiency [57].

Dengue viruses employ a sophisticated strategy by activating global autophagy to generate resources for replication while concurrently blocking mitophagy. This specific inhibition of mitochondrial clearance ensures that damaged mitochondria, which may serve as viral replication platforms, are not eliminated [49]. DENV hijacks the cell’s general autophagy pathway to create a favorable environment for autophagosomes, which are exploited to provide scaffolds for viral replication complexes and to generate nutrients from degraded host components [58,59].

On the other hand, DENV employs precise strategies to inhibit mitophagy by disrupting the PTEN-induced kinase 1 (PINK1)–Parkin pathway and modulating mitophagy receptors. To disable the PINK1–Parkin pathway, DENV infection prevents the stabilization of PINK1 on damaged mitochondria and blocks the subsequent translocation of Parkin. This functional blockade is achieved through the virus-induced downregulation of both PINK1 and Parkin proteins, effectively crippling the cell’s ability to initiate mitophagy. To ensure a complete shutdown of mitochondrial quality control, DENV also inhibits receptor-mediated mitophagy. This prevents the cell from using alternative, Parkin-independent pathways to clear damaged mitochondria, thereby exacerbating cellular injury [24].

To maintain a healthy organelle population, cells balance the creation of new mitochondria (biogenesis) with the removal of old ones (mitophagy) [60,61]. DENV systematically dismantles both critical quality control arms. Paradoxically, despite causing severe mitochondrial injury that normally triggers a compensatory response, DENV infection actively suppresses mitochondrial biogenesis. The virus achieves this by downregulating the expression of master regulators like PGC-1α and nuclear factor erythroid 2-related factor 2 (NRF2). By crippling this renewal pathway, DENV prevents the cell from replacing damaged organelles, leading to reduced mitochondrial turnover and a decline in functional mitochondrial capacity [24,62].

## 4. DENV Hijacking of Host Cell Metabolism via Mitochondrial Modulation

Dengue belongs to a perplexing interaction with host cells, mainly affecting mitochondria, which contribute to cellular metabolism, cell homeostasis, autophagy, and innate immunity. DENV proteins manipulate host cell metabolism to establish a pro-viral state that facilitates replication and immune evasion. This metabolic takeover ensures a continuous supply of energy and biosynthetic precursors essential for the viral lifecycle [23,63,64]. Dengue viral NS1 protein is a metabolic modulator, particularly in cellular energy metabolism, thereby increasing glycolytic flux, which promotes shifting metabolism from OXPHOS to glycolysis. By that, NS1 triggers MMP loss and decreases ATP production, and disrupts cell innate immunity pathways [52,65,66].

Meanwhile, NS3 protein is a key viral effector that localizes to mitochondria, directly sabotaging cellular bioenergetics. The primary function of NS3 protein at the mitochondria is to impair the electron transport chain by inhibiting respiratory complex 1 (CI). This disruption is dependent on the proteolytic activity of the NS3 protease (NS3pro) domain. Both full-length NS3 and NS3pro have been shown to decrease mitochondrial respiration by specifically targeting CI’s NADH; ubiquinone oxidoreductase activity, while a catalytically inactive version of the protease, has no effect. This strongly suggests that NS3 acts by cleaving subunits of CI, thereby disrupting the electron flow necessary for ATP synthesis. By impairing mitochondrial respiration, NS3 plays a crucial role in altering the host’s cellular metabolism and can contribute to increased oxidative stress [67,68,69].

Hydrophobic membrane protein NS4A and NS4B are correlated with the disruption of mitochondria associated-membranes (MAMs) and induction of maximum autophagy, interfering in IFN-1 production followed by antagonism in immune response. This way, the NS4A and NS4A-NS4B complex incite the cell membrane remodel and disrupt the clearance of damaged mitochondria by inhibiting fragmentation with elongated mitochondria [44,70,71]. Therefore, the crucial structural capsid proteins are directly connected with lipid droplets (LDs), which are important for energy storage and viral assembly [72,73].

## 5. DENV Evasion of Innate Immunity

Dengue virus actively subverts the host’s innate immune system to create an environment conducive to its replication and survival. This involves hijacking key cellular machinery and directly targeting immune signaling pathways to neutralize antiviral defenses. MAVS protein functions as the primary signaling platform for the innate immune response against RNA viruses like DENV. Located on the outer mitochondrial membrane, MAVS protein is activated upon sensing viral RNA, initiating a cascade that leads to interferon production. Because of this vital role, MAVS is the primary target for DENV’s immune evasion strategies. DENV disrupts the MAVS signaling cascade along with hindering the activation of downstream molecules like TNF receptor-associated factors 2, 3, and 6 (TRAF2/3/6) and TANK-binding kinase 1 (TBK1), resulting in the suppression of interferon (IFN) responses [74,75,76].

DENV follows some strategies notably via the functions of NS2A/B, NS3, and NS4A/B proteins, which lead to antagonism of the IFN system and circumvent innate immunity. NS2B/3 protease complex promptly disrupts the RIG-I/TLR-3 signaling pathway by degrading interferon regulatory factor 3 (IRF3) and stimulator of interferon genes (STING) which leads to blocking IFN-β transcription for inflammatory response. NS3 protein impairs the function of MAVS protein by altering the mitochondrial membrane integrity, resulting in the dislocation of retinoic acid-inducible gene I (RIG-I) to the adaptor MAVS. Thereby, targeted MAVS availability on behalf of dengue infection leads to lowering IFN signals [25,74,77,78]. Therefore, NS4A and NS4B are responsible for the alteration of MMP and ER-mitochondria contact sites which favor blocking upstream RIG-I activation by inhibiting its ATPase activity and suppressing TBK1 and IRF3 phosphorylation. As a result, interruption with signal transduction beyond MAVS and declination of nuclear translocation of IRF3 occur, resulting in no IFN gene activation [74,79].

## 6. Immune-Mediated Mitochondrial Alterations: The Hidden Cost of Antibody Dependent Enhancement (ADE) in Dengue

**Figure 2:**
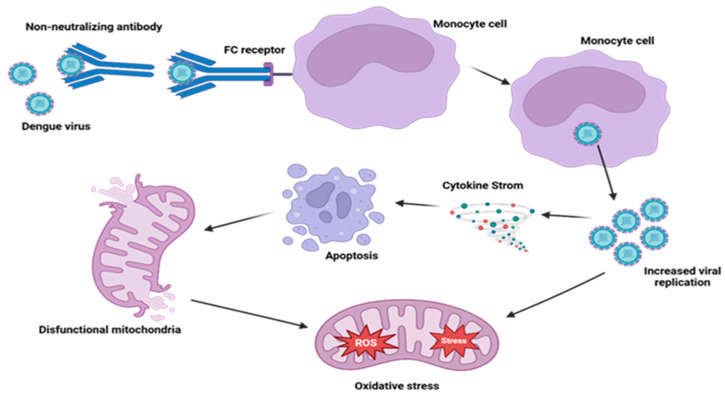
ADE induces mitochondrial dysfunction and inflammation in dengue-infected cells. DENV entry into monocytes is stimulated by non-neutralizing antibodies along with Fc receptor-mediated uptake, enhancing viral replication. This ensues cytokine storm, oxidative stress due to increased reactive oxygen species (ROS), and mitochondrial dysfunction that leads to apoptosis and once more triggers the inflammatory response, worsening disease severity.

DENV infection by one serotype assures a long-lasting immunity against that homogenous serotype while it confers only minimal and ephemeral protection against subsequent infections with heterogenous DENV serotypes [80]. ADE is a phenomenon in which pre-existing, sub-neutralizing antibodies increase the risk of severe disease during a subsequent infection with a heterologous DENV serotype. This occurs because the antibodies bind to the virus to form immune complexes that, instead of neutralizing the infection, facilitate viral entry into host cells (such as monocytes, dendritic cells, and macrophages) via their interaction with Fcγ receptors (FcγR). Between the two types of ADE, intrinsic ADE plays a greater role in elevating dengue replication by inhibition of type 1 interferon and activation of interleukin-10 (IL-10) biosynthesis, whilst extrinsic ADE helps to facilitate virus entry. Although ADE has a recognized role in exacerbating viral replication and immune dysregulation, recent research indicates its significant contribution to mitochondrial dysfunction [81,82,83,84]. ADE-induced enhanced viral load and hyperactivation of immune cells leads to elevated production of inflammatory cytokines along with ROS and NO, resulting in the exertion of oxidative stress on mitochondria (Figure 2). This intensifying oxidative stress alters the MMP, disrupts mitochondrial energy production, and metabolic pathways [85,86,87].

## 7. Mitochondrial Dysfunction, Oxidative Stress, and Apoptosis in DENV Infection

Dengue infection creates a hostile environment in cells, leading to direct mitochondrial damage both structurally and functionally, playing a critical role in viral pathogenesis and immune evasion. Among the several DENV-induced mitochondrial injuries, mitochondrial structural damage and membrane depolarization cause severe mitochondrial dysfunctions along with excessive mitochondrial ROS accumulation, MAVS complex disruption, and apoptosis [23,88]. DENV infection enhances mitochondrial elongation by inhibiting Drp1 phosphorylation and suppressing MFN1/2 and optic atrophy 1 (OPA1), leading to fragmented mitochondria that are responsible for impaired electron transport chain (ETC) function along with reduced OXPHOS and ATP generation [52,53,67].

Impaired mitochondrial ETC in dengue-infected cells enhances ROS production that leads to oxidative damage in mitochondrial lipids, proteins, as well as mitochondrial membranes, and DNA. The adverse effects of the dengue virus on respiratory properties and mitochondrial membrane depolarization alleviate the efficiency of ATP synthesis, indicating the rising energy demand followed by the synthesis of dengue viral proteins. Thus, DENV proteins interact with the mitochondrial membrane and affect the MMP and its permeability along with increasing proton leak, which leads to the formation of superoxide (O_2^−^_) and other ROS.

Consequently, increased oxidative stress emerges, thereby elevating ROS accumulation during dengue infection. In addition, DENV-induced fragmented mitochondria enhance the release of mitochondrial pro-apoptotic factors like cytochrome, which causes severe cell death [67,89,90]. DENV induces apoptosis or programmed cell death by activating both intrinsic (mitochondrial) and extrinsic (death receptor) apoptotic pathways. Mitochondrial membrane permeabilization and activation of cell surface death receptors by dengue infection permits the overall apoptosis process followed by apoptosome formation [26].

## 8. Mitochondria Targeted Therapeutic Implications for Dengue: Emerging Insights and Drug Candidates

There is currently no specific antiviral medication to treat dengue fever. The management of the illness primarily focuses on supportive care to relieve symptoms and prevent complications. Mitochondria, which play a central role in cellular metabolism, immune signaling, and apoptosis, can be a potential therapeutic target in disease conditions like dengue or dengue-related liver disease, cancer, and other metabolic diseases [91,92].

Mitochondrial dysfunction beyond DENV infection such as disrupted membrane potential, excessive ROS generation, impaired OXPHOS, and altered mitochondrial dynamics, contributes to viral replication, immune evasion, and disease severity [79,90,93]. The above-mentioned mitochondrial dysfunctions are related not only to dengue, but also aging, cancer, and age-related neurodegenerative and metabolic syndrome [94]. Targeting these mitochondrial vulnerabilities can be an adequate strategy to minimize dengue pathogenesis along with mitochondrial dysfunction [95].

Recent research has shown that several compounds are able to modulate mitochondrial dynamics. Compounds that modulate protein oligomerization, inhibit guanosine triphosphatase (GTPase), and regulate the transcription of the fusion/fission machinery have therapeutic potential against mitochondrial dysfunction-related diseases [96]. Mitochondrial activity stimulation molecule 7 (MASM7) is the activator of MFNs protein oligomerization that can improve mitochondrial activity by increasing MMP, respiration, and ATP production. Although MASM7 is a preclinical experimental compound, it can be used in severe dengue to enhance endothelial cell bioenergetics to resist plasma-leak by rescuing the balance fusion of mitochondria. The possible administrative route of MASM7 would be systemic as it is bioavailable for nanoparticle delivery in an animal model [97,98,99].

In the meantime, the compound mitochondrial division inhibitor-1 (mdivi-1) inhibits GTP hydrolysis related to the mechanoenzymes liable for shaping mitochondrial morphology and the GTPase activity of these proteins (OPA1, MFNs and DRP1) and can be a potential target for modern therapeutic intervention to protect mitochondrial function in many disease conditions like stroke, myocardial infarction, and neurodegenerative diseases [100].

Several antioxidants like melatonin, N-acetylcysteine (NAC), Mito-Q, and SkQ-1 can play an important role in rescuing mitochondrial functionality via downstream on mitochondrial ROS and redox balance. Melatonin has mitochondrial-protective effects like sirtuin 1 (SIRT1) and autophagy modulation along with several in vitro reports of dose-dependent suppression of dengue replication across serotypes. It is orally available with a low risk that makes it a prominent candidate for adjunctive trials [101,102,103,104,105]. NAC is an antioxidant that can reduce oxidative stress by increasing plasma antioxidants like glutathione peroxidase and glutathione reductase in severe dengue-induced liver failure. NAC can be used in the treatment of acute dengue-associated liver disease via intravenous administration [106,107]. NAC was approved by the food and drug administration (FDA) for several uses, including an antidote for acetaminophen overdoses and as a mucolytic agent to break down mucus in chronic respiratory conditions like chronic obstructive pulmonary disease and cystic fibrosis [108,109,110,111]. NAC is also available as a dietary supplement like melatonin and Mito-Q. They are already widely available as over-the-counter dietary supplements, which confirm their oral bioavailability and general safety in humans.

Compounds that modulate mitochondrial function have both prophylactic and therapeutic potential, by creating a host environment non-permissive to DENV by countering the active dengue infection. Prophylactic strategies against dengue can alter the host’s cellular environment to make it less permissive to viral infection or replication upon exposure. NAC, Mito-Q, and SkQ-1 are available dietary supplements; they are suitable for chronic administration to maintain a heightened state of mitochondrial protection. Moreover, an oral formulation would be most suitable for prophylactic use. Their unique chemical structure allows them to accumulate to supraphysiological levels within the mitochondrial matrix, providing potent antioxidant defense at the primary source of DENV-induced ROS [112,113,114]. By maintaining mitochondrial resilience, these compounds could theoretically raise the threshold of cellular stress required for DENV to establish a productive infection and trigger downstream pathogenesis.

A therapeutic agent, such as NAC, was used during an active infection to reduce DENV viral load, mitigate symptoms, and prevent the progression to severe disease. Multiple case reports have documented the successful use of intravenous NAC to treat patients with dengue-associated acute liver failure [106,107,115]. In these cases, IV administration led to a rapid decrease in liver transaminases, normalization of coagulation profiles, and favorable clinical outcomes, even in patients with poor prognostic factors. An intravenous route would be necessary for therapeutic intervention in hospitalized patients with severe disease, though further studies are needed. MASM7 may have therapeutic potential. DENV protease actively cleaves MFN proteins to inhibit mitochondrial fusion, a process that disrupts cellular bioenergetics [52]. The vascular leakage that characterizes severe dengue is fundamentally a consequence of endothelial cell dysfunction and energy failure. By pharmacologically activating MFNs, MASM7 could directly counteract this viral strategy, restore the integrity of the mitochondrial network, improve endothelial cell bioenergetics, and thereby enhance the resilience of the vascular endothelium against plasma leakage. In this regard, MASM7 can be a potential host-directed therapy specifically for preventing or treating the vascular permeability associated with severe dengue.

Despite known connections between dengue and mitochondrial dysfunction, the potential of compounds that target this dysfunction as a viable anti-dengue therapy are not well-investigated. The established link between dengue pathogenesis and severe mitochondrial stress provides a rationale for repurposing these drugs to restore cellular bioenergetics, reduce oxidative damage, and ultimately mitigate disease severity. Therefore, despite compelling preclinical evidence in other disease models, dedicated research is needed to validate these mitochondrial-targeting agents as a viable, host-directed therapeutic strategy for dengue fever.

## 9. Future Direction

Recent research demonstrates that DENV drastically alters mitochondrial structure and function through a coordinated attack: it impairs cellular bioenergetics, disrupts quality control mechanisms like mitophagy and biogenesis, and simultaneously subverts innate immunity by dismantling the MAVS signaling pathway. Although current studies have provided much insight, there is still a lack of clarity about mt-DNA-mediated inflammation during DENV infection, cell-type-specific interactions with the dengue virus, and the potential value of the specific molecules as a therapeutic target. Future investigations are needed to define the potential of specific proteins like NS4A and MAVS as therapeutic targets and cell type-specific effects of DENV proteins, particularly in immune cells, endothelial cells, and neurons.

Further, there is limited research on host-targeted compounds, having specific restoring capacity of mitochondrial homeostasis and inhibiting viral replication, along with fission/fusion modulators, mitochondrial antioxidants, and mitophagy inducers [101,116,117].

## 10. Conclusions

Dengue viruses pose a major public health problem by significantly influencing host cell functions through the disruption of mitochondria, which are the central organelles regulating cellular energy metabolism, innate immunity, and apoptosis. Recent research demonstrates that this attack is comprehensive, simultaneously disrupting cellular bioenergetics, impairing mitochondrial dynamics, dismantling quality control pathways like mitophagy and biogenesis, and triggering oxidative stress. Additionally, DENV proteins, particularly NS2B/3, NS4A/B, and capsid, interacts with MAVS, favoring viral replication through evading host innate immune responses. This disrupted cellular processes and mitochondrial function leads to severe consequences as well as disease conditions. This review covers the broad aspects of DENV infection-induced altered mitochondrial homeostasis and functions along with its consequences and different therapeutic implications. An extended investigation on dissecting the exact signaling pathways, cell-type specific mitochondrial responses, and identification of mitochondrial-targeted therapeutics could mitigate DENV pathogenesis. Therefore, continued investigation into this intricate virus–host interplay and translating these molecular insights into effective clinical strategies will be crucial in the global fight against this pervasive pathogen are essential.

## Figures and Tables

**Table 1:** Mitochondrial dysfunctions correlated with dengue viral infection.

Aspect	Dengue Viral Infection	Mitochondrial Dysfunctions	Ref.
Mitophagy	Modulates mitophagy to promote viral replication	Impaired mitophagy fails to clear damaged mitochondria	[24]
Innate Immune Response	Inhibits mitochondrial antiviral signaling (MAVS) protein to evade immune detection	MAVS dysfunction leads to impaired antiviral responses	[25]
Apoptosis Pathways	Activates intrinsic apoptosis pathways	Leads to cell death and tissue damage	[26]
Energy Production	Impairs mitochondrial oxidative phosphorylation	Leads to reduced Adenosine triphosphate (ATP) production	[27]
Oxidative stress	Increases reactive oxygen species (ROS) levels due to viral-induced stress	Excessive ROS damages mitochondrial DNA (mt-DNA) and proteins	[28,29]
Mitochondrial membrane potential (MMP)	Reduces MMP (Δψm)	Loss of MMP (Δψm) disrupts mitochondrial integrity	[30,31]
Calcium Homeostasis	Causes mitochondrial calcium overload	Impairs calcium signaling and buffering capacity	[32]
mt-DNA	Causes mt-DNA damage through oxidative stress	mt-DNA mutations disrupt mitochondrial function	[33]
Inflammatory Response	Triggers cytokine production (e.g., IL-6, TNF-α)	Inflammation is both a cause and consequence of dysfunction	[34]

## Abbreviations

The following abbreviations are used in this manuscript:

DENV	Dengue virus
DHF	Dengue hemorrhagic fever
DF	Dengue fever
DHS	Dengue hemorrhagic syndrome
DSS	Dengue shock syndrome
ATP	Adenosine triphosphate
ROS	Reactive oxygen species
ETC	Electron transport chain
OXPHOS	Oxidative phosphorylation
mtDNA	Mitochondrial DNA
DRP1	Dynamin related protein 1
DNM2	Dynamin 2
OMM	Outer mitochondrial membrane
IMM	Inner mitochondrial membrane
MMP	Mitochondrial membrane potential
FcγR	Fragment crystallizable gamma receptors
MAVS	Mitochondrial antiviral signaling
AMPK	AMP-activated protein kinase
MASM7	Mitochondrial activity stimulation molecule 7
ADE	Antibody dependent enhancement
GSH	Glutathione
MFN1	Mitofusin 1
MFN2	Mitofusin 2
FIS1	Fission 1
OPA1	Optic atrophy 1
PGC-1α	Peroxisome proliferator-activated receptor gamma coactivator 1-alpha
O_2^−^_	Superoxide
TNF-α	Tumor necrosis factor-alpha
IL-10	Interleukin-10
IFN1	Type 1 interferon
NO	Nitric oxide
CI	Complex 1
NS3pro	NS3 protease
LDs	Lipid droplets
NRF-2	Nuclear factor erythroid 2-related factor 2
PPAR-γ	Peroxisome proliferator-activated receptor gamma
RIG-1	Retinoic acid-inducible gene I
IRF3	Interferon regulatory factor 3
TRAF 2/3/6	TNF receptor-associated factors 2, 3, and 6
STING	Stimulator of interferon genes.
UCP1	Uncoupling protein 1
PINK1	PTEN-induced kinase 1
Ser 616	Serine 616
TBK1	TANK-binding kinase 1
MAMs	Mitochondria-associated membranes
Sirtuin 1	SIRT1
Mdivi-1	Mitochondrial division inhibitor-1
NAC	N-acetylcysteine
FDA	Food and drug administration
